# The implications of the Sudan war on healthcare workers and facilities: a health system tragedy

**DOI:** 10.1186/s13031-024-00581-w

**Published:** 2024-03-17

**Authors:** Rawa Badri, Iyas Dawood

**Affiliations:** 1 Mycetoma Research Centre, Khartoum, Sudan; 2 Faculty of Medicine, University of Khartoum, Khartoum, Sudan; 3 Fauclty of Medicine, Omdurman Islamic University, Khartoum, Sudan

**Keywords:** Sudan war, Health system, Attacks, Healthcare workers, Facilities

## Abstract

In light of a collapsing healthcare system in Sudan, attacks on healthcare institutions and staff have markedly increased since the eruption of war on the 15th of April, costing many precious lives and valuable hospitals. Around 60 attacks on health facilities have been reported so far, many occupied by one or the other sides of the conflict, and the rest exhibit medication shortages and safety issues; hence, two-thirds of the medical centers are nonfunctioning. More than 200 violations against medical staff were recorded all over the country, which led to the death of 38 healthcare workers. Killing, kidnapping, and assaulting doctors, consequently led to a huge shortage in staff in the few barely working facilities, as the remaining health workers were concerned regarding their safety. Recommendations consisted of ceasing fire, restoring and resuming healthcare services, and insurance of a safe working environment. International collaboration and sufficient financial support are crucial to restore the healthcare system in Sudan.

This year-2023 has witnessed numerous instances of armed conflicts across the globe, healthcare facilities are often targeted in armed conflicts, particularly in urban areas, leading to further destruction of the healthcare system and hospitals in many countries [1].

The Sudanese government has a history of systematically targeting doctors, as evidenced by the case of Dr. Ali Fadul Ahmed who was killed in April 1990 after enduring torture by the National Intelligence and Security Service (NISS) in the aftermath of Omar al-Bashir's coup. Since the onset of civil unrest in Sudan in December 2018, medical professionals have been subjected to police brutality for providing care to victims of government violence. Dr. Babiker Abdel Hamid and Mahjoub Eltaj are among the individuals singled out, with many doctors being held in substandard detention facilities. Past incidents have seen doctors disappear, including five in 2009 and two in 2017 from Kalma IDP camp, allegedly abducted by pro-government militias for treating individuals injured by security forces [2].

In 2021, Sudan experienced a notable rise in violent occurrences impacting healthcare, with 52 incidents documented compared to 15 in 2020. The healthcare system was immediately affected following the October 2021 coup, leading to the tragic loss of two physicians during a demonstration in Khartoum. Over the course of the year, there were 35 protest-related events affecting healthcare services, primarily concentrated in Khartoum state but also extending to other areas. Healthcare professionals, encompassing doctors, psychologists, and nurses, encountered injuries and apprehensions during these incidents. On November 17, the most fatal day of the protests, reports indicated that 15 doctors were detained at medical facilities in Khartoum and Omdurman. Alarmingly, there were accounts of female doctors being subjected to sexual assault by law enforcement officers. State security forces utilized tear gas within hospitals from October 25 to December 31, 2021, with the emergency department of Khartoum Teaching Hospital being targeted on three occasions, resulting in respiratory complications for healthcare staff and patients [3]. After the military coup that happened in October 2021, political violence has continued to impact health workers and facilities, with a 69% increase in incidents in 2022 compared to the previous year [4].

Since the conflict began between the Sudanese Armed Forces and the Rapid Support Forces in mid-April 2023, approximately 6.6 million individuals have been forced to leave their homes within and outside of Sudan. The death toll has surpassed 12,190 since the fighting erupted [5].

This paper provides an overview of the assaults by the Rapid Support Forces and the Sudanese Armed Forces on healthcare facilities and personnel from the onset of the armed conflict in mid-April to mid-December 2023.

## Destruction of healthcare facilities

In Sudan, many brutal actions done by unauthorized armed forces have been reported in West Sudan, and healthcare institutions were damaged, corrupted, and occupied by militias in Darfur [6].

Six months after the start of the conflict in Khartoum on the 15th of April, the WHO Regional Director of the Eastern Mediterranean has stated that 70% of Hospitals and medical centers are out of service and 20% of medical institutions far from engagement areas are severely affected [7]. According to the most recent WHO statistics that were revealed on the 31st of October at the UN agencies conference, 60 attacks on healthcare facilities have been verified in the conflict areas, leading to the death of 34 people and injuring 38 [8]. Secondary and tertiary Hospitals are considered to be valuable components of the country’s infrastructure, regrettably, many were violated, destroyed, and bombed either by airstrikes or artillery, and what happened Ibn Sina Hospital while medics were working [9]. “Days after evacuating the hospital, we were surprised by some patients were returning to it without our knowledge, to get oxygen,” said Elnimer Jibreel the General Director of Al Shaab Hospital, “They stayed there for 48 h until a shell fell directly on the hospital, and we evacuated them again”[10]. Furthermore, the effects and clues regarding strikes on Ibn Sina Specialized Hospital and Al Roumi Medical Center were well documented.

Nine hospitals were confirmed to be occupied by involved sides of the conflict, bringing destruction and obstruction [9]. Involvement of health care facilities witnessed to be done with intent, the RFS were settling armed soldiers, vehicles, and anti-aircraft weapons around East Nile Hospital, and as a result, the hospital and its surroundings were turned into battlefields. The hospital was targeted on the 1st of May by an airstrike.

Ambulances were targeted in many incidents, affecting not only patients’ mobilization but also oxygen and fuel delivery [11]. On August 14, 2023, in Khartoum, six ambulances were attacked by the military forces, preventing the transportation of patients [12]. Additionally, the Sudanese Armed Forces (SAF) bombed the Doctors Hospital in Khartoum, resulting in the destruction of the building and its medical equipment [13]. Those few Hospitals that survived strikes and occupation are under electricity blackouts, and out of fuel for electrical generators [11], as a result, in Eld’aeen Hospital in East Darfur, it has been estimated that 30 newborns have died because of issues related to electricity and oxygen.

The ongoing conflict in Sudan is jeopardizing the work of important medical institutions like the Sudanese Childhood Diabetes Association, as healthcare facilities are being targeted and essential medicines, such as insulin, are being damaged or depleted [14]. Moreover, the Mycetoma Research Centre (MRC) in Khartoum, the only WHO collaborating center dedicated to mycetoma management, has stopped operating. As a result, many patients have been unable to receive treatment since the war began. The center is currently inaccessible and has suffered some damage [15].

## Attack on healthcare workers

Since the begining of the ongoing armed conflict, Insecurity Insight has recorded at least 222 assaults on the healthcare system in Sudan between January 1st to October 31st.These attacks have resulted in the deaths of 38 healthcare workers and the damage of health facilities at least 49 times. These incidents have significantly hindered healthcare providers' capacity to adequately serve patients, maintain sufficient staffing levels, and have also affected the public's access to healthcare [16]. The rising violence against healthcare workers in Khartoum is posing a threat to the limited number of hospitals that are still operational in the Sudanese capital, according to Médecins Sans Frontières (MSF). This concern arose after 18 MSF staff members were physically assaulted by armed individuals while assisting the Turkish Hospital in Khartoum [17, 18]. Numerous incidents, reportedly involving RSF fighters, have occurred in Darfur and Khartoum, leading to the closure of medical facilities and disruptions in the supply of essential medicines from the capital to Darfur [16].

The Shaheed Hospital in the Drushab suburb of Khartoum North City, Khartoum state, was reportedly attacked by RSF soldiers on June 30, 2023. The attack resulted in the death of a laboratory specialist and the assault of patients, as well as the destruction of the hospital's laboratory. Additionally, four doctors and a pharmacist were allegedly killed. On July 3, 2023, in Khartoum state, an RSF soldier reportedly killed a second-year student et al. Hayat University's Faculty of Medical Laboratories. Then, on July 4, 2023, in Omdurman City, Khartoum state, a Health Ministry employee was fatally shot inside the MSF-supported Saudi Maternity Hospital, leading to the hospital's closure and staff seeking a safer location to continue providing services [19].

On September 6, 2023, three doctors and five other individuals were detained at an RSF base near Teiba Camp in Jebel Aulia village, Khartoum state, while they were on their way to volunteer et al. Ban Jadeed Hospital in the East Nile suburb of Khartoum, which is the only operational hospital in the area. Then, on September 12, 2023, the upper floor of the Weapon Medical Hospital in Nyala City, South Darfur State, was struck by a shell of unknown origin. On September 13, 2023, a hospital director was reportedly abducted by the RSF in Khartoum–Al Azhari, Khartoum city and state, from a hospital building possibly and has been released on an unspecified date [20].

A medical strike occurred in retaliation for the shooting of the medical director of Bashayer Hospital and a member of the Southern Khartoum Belt Emergency Monitoring Room by an RSF member, who also threatened the rest of the medical team at the hospital. Bashayer Hospital provides healthcare services to residents of several neighborhoods in Khartoum. This incident reflects the worsening security situation in the Sudanese capital, leading to the closure of most hospitals in Khartoum due to being caught in the crossfire or occupied by the RSF [21].

Local reports indicate that on November 24, medical workers at El Obeid Teaching Hospital ceased their duties in response to an altercation in which a female doctor was assaulted by military personnel. As a result, hospital operations have come to a standstill, disproportionately affecting patients who cannot afford private clinic expenses [22].

Healthcare workers are facing dangers from armed groups as well as the general chaos and criminal activity caused by political instability. For example, Dr. Bushra Mohammed Ibnauf Sulieman was tragically killed in his own home, possibly by robbers, as reported by his friends. He was a gastroenterologist and co-founder of the Sudanese American Medical Association [23]. In El Geneina, it is reported that RSF fighters entered the Medical Rescue Center clinic and lethally shot Dr. Adam Zakaria Is'haq, who was administering medical treatment at the clinic following the destruction of the main hospital by armed militia and RSF in late April, along with 13 patients, as documented by witness testimonies gathered by Amnesty International [24]. Moreover, eyewitnesses reported that a prominent doctor, Dr Abdel Moneim Banga Abdel Hafeez, was assassinated by Sudanese army forces in Omdurman on January 17, 2024. The doctor was accused of collaborating with the paramilitary Rapid Support Forces (RSF), although his family denied this claim and stated that he was killed by a bullet from an unknown source while residing in an area controlled by the RSF [25].

In addition to the fatalities, 4 healthcare workers have been taken captive by militia and 9 others are missing. Hospitals that are still operational are facing a critical shortage of medical staff. Many healthcare workers have fled the capital since the conflict began, greatly reducing the capacity of hospitals. The remaining medical staff are either too afraid to go to work due to safety concerns or are overworked and struggling with a lack of specialized professionals such as surgeons and anesthetists, as well as a shortage of medical supplies [26].

## Implications on health services

Inevitably, patients with chronic diseases struggled with the conflict. 8,000 patients who were receiving regular sessions of dialysis were affected by the conflict, some died because of electricity blackouts -as those in Al-obaid, and some were forced to reduce their session time and frequency. Dialysis centers in the affected areas were lost and the rest in other cities have become overcrowded due to the turnout of displaced people [27]. At present, more than 9000 people need dialysis in Sudan.

The latest updates regarding epidemics were worrying, people who were at risk of cholera exceeded 3.1 million by the previous December 2023, with 6939 suspected cases and 200 deaths as recorded by the WHO. 4557 cases of Measles have been recorded with 105 deaths, and Dengue fever incidence was markedly escalating as well. Moreover, 4.2 million women and children were recognized to be at risk of gender-based violence. Other problems related to medication shortages -especially paediatric insulin, and an increase in unsafe home deliveries were well documented [28]. With the intense conflict, major hospitals and centers were severely affected. Only three out of eight cardiac centers are functioning at the beginning of 2024. The country has lost three oncology centers since the start of the war, one of them was providing care for over 60% of cancer patients.

In the absence of a stable political situation in Sudan, considerable regional efforts rose to assist health system dynamics. The resistance committees have played an important role in providing supplies to the medics, assisting in transferring patients, and rehabilitating healthcare centers to ensure the continuation of medical care services [29]. Many integrated attempts between civil society representatives and international agencies to guarantee health workers’ safety have been made. In the “Jeddah Declaration of Commitment” on the 11th of May 2023, both sides of the conflict committed to seven points focused on protecting civilians as the major purpose and making safe passage to humanitarian assistance. However, a lot of violations have been recorded after the Jeddah talk, showing no commitment from both factions [30].

## Recommendations

Halting the current conflict in Sudan is of utmost importance, as it has led to the displacement of millions and disrupted the healthcare system. It is essential to adhere to international humanitarian laws to protect civilians and infrastructure [15]. International health organizations' rule is crucial in restoring and sustaining vital healthcare services, including supplying necessary resources to healthcare facilities, providing treatment for infectious and chronic diseases, and resuming routine health programs. It is crucial to address the crisis in Sudan and provide assistance to the healthcare system during and after the conflict [14, 15]. The WHO and UNICEF in collaboration with the Federal Ministry of Health are highly encouraged to focus on rehabilitating primary healthcare centers, especially in the conflict areas. This will help early detection and prevention of infectious diseases and malnutrition problems, provide the required vaccines, and help receive psychological treatment.

Following the start of the conflict, the United Nations Population Fund (UNPFA) and the National Medical Supply Fund (NMSF) local centers and supplies were occupied by the military [11]. Funding from the United Nations and other human rights organizations is immensely needed at present. Sufficient financial support will assist in rebuilding important centers such as heart and oncology centers, bringing life-saving equipment and machines including dialysis machines, enhanced security for healthcare facilities and personnel, and increased availability of medical supplies are essential to mitigate the impact of the conflict and deliver crucial healthcare services to those in need [15].

Furthermore, sustaining and maintainng the health system in Sudan encompassed by many challenges. The absence of a well-recognized government in Sudan to tackle the international agencies and the unsafe work environment for medics and health organizations are the main issues. Healthcare workers are working under dangerous conditions and must be safeguarded to guarantee the continuity of healthcare services during this emergency [15].

## Data Availability

Not applicable.
